# How Internal Marketing Drives Employees’ Internal Relationship Quality of Service Organizations Between Mainland China and Taiwan: The Moderating Roles of Internal Relationship Investment and Leader-Member Exchange

**DOI:** 10.3389/fpsyg.2021.794492

**Published:** 2021-12-16

**Authors:** Yunhe Li, Minghua Xiong, Wei-Hsuan Chang, Ling Li

**Affiliations:** ^1^College of Economics and Management, Shangqiu Normal University, Shangqiu, China; ^2^School of Economics & Management, Foshan University, Foshan, China; ^3^College of Attainment, Nanfang College Guangzhou, Guangzhou, China; ^4^Research Center of the Economic and Social Development of Henan East Provincial Joint, Shangqiu Normal University, Shangqiu, China

**Keywords:** cultural comparison, internal marketing, internal relationship investment, internal relationship quality, internal service recovery, LMX

## Abstract

Recently, issues of human resource management gradually attract a lot of attention from organizational behavior scholars, thus how to effectively improve service employees’ job attitude and performance to meet the needs of stakeholders is one of the key issues in internal marketing. Based on the perspective of internal marketing, the study transforms the relevant factors applied to maintaining external customer relations into internal employee-oriented factors, so as to increase the understanding of the relationship between internal service recovery and internal relationship quality (IRQ). This study aims to explore (1) whether internal service recovery enhances IRQ; (2) whether internal relationship investment (IRI) positively moderates relationship between internal service recovery and IRQ; and (3) whether effectiveness of internal service recovery differentiates under different exchange relationship (high/low quality leader-member exchange). In this study, a total of 206 Mainland China and 250 Taiwanese participants were collected. In this study, a variance-based structural equation modeling (PLS-SEM) was performed to test the proposed hypothesizes and conduct comparative analysis. Empirical results in both samples show that internal service recovery has positive and significant effects on IRQ; internal relationship investment and leader-member exchange (LMX) positively and significantly moderate the relationship between internal service recovery and IRQ. Finally, based on the results, this study provides some discussions, suggestions and managerial implications for future studies in organizational management.

## Introduction

Nowadays, in the highly competitive environment, service is playing a vital role in gaining competitive advantage for organizations (Harun et al., 2018; Hewagama et al., 2019). Many service organizations are also constantly exploring ways to improve service quality, so as to create higher customer loyalty and satisfaction to establish good customer relations (Van Vaerenbergh and Orsingher, 2016; Van Vaerenbergh et al., 2019). Service failure may occur during service delivery, as long as it occurs below customers’ requirements or even does not meet their requirements, then there will be service failure (Harun et al., 2018; Van Vaerenbergh et al., 2019). However, service is provided by front-line employees who directly face customers, and the service quality perceived by customers depends on the current behavior and attitude from front-line employees, and affects customers’ feelings of receiving service (Van Vaerenbergh and Orsingher, 2016; Harrison-Walker, 2019; Hewagama et al., 2019; Mihardjo et al., 2020). If front-line employees are influenced by negative factors at work while providing services, they will have cognitive dissonance, which may lead to a failure in the service provided (Hewagama et al., 2019; Huang, 2020). Therefore, whether a service organization is provided with appropriate remedial strategy and high-quality staff has become a key factor for the success (Van Vaerenbergh and Orsingher, 2016; Park and Tran, 2018; Harrison-Walker, 2019). Thereinto, the internal relationship quality (IRQ) plays a vital role in enhancing employees’ sense of identity and centripetal force to the organization. internal relationship quality IRQ means a series of positive attitudes and psychological states that employees perceive the importance given by the organization, and high IRQ contributes to enhancing employees’ loyalty to the organization and reduce negative emotions and behaviors.

With the evolution of marketing concept, the target of marketing has been gradually extended from external consumers and firms to internal customers in organizations, thus producing the concept of internal marketing (Hewagama et al., 2019; Huang, 2020; Mihardjo et al., 2020). To gain competitive advantage in the service market, organizations necessarily first meet the needs and desires of employees, and then employees will provide better services to customers (Harrison-Walker, 2019; Huang, 2020; Mihardjo et al., 2020). There have been relevant studies on internal marketing for decades, which emphasize that organizations ought to treat employees as internal customers and provide work that meets their needs (Joung et al., 2015; Park and Tran, 2018; Huang, 2020), However, there is little discussion about how organizations should support internal customers in dealing with service failure (Yoo et al., 2006; Hewagama et al., 2019; Mihardjo et al., 2020). Most of the previous studies on service recovery were conducted from the view of customers, and few of them discussed from the view of front-line employees how to remedy the helplessness produced by employees after they experience service failure (Bowen and Johnston, 1999; Yoo et al., 2006; Lambert et al., 2016; Hewagama et al., 2019). The main purpose for Internal Service Recovery (ISR) is to make employees more confident while providing services to external customers and make sure they feel that the organization is trustworthy and satisfied with the work provided by the organization (Park and Tran, 2018; Huang, 2020). This study aims to explore the impact of internal service recovery on maintaining and improving the relationship between organizations and employees.

Besides, to maintain and improve the close relationships among external customers, organizations will attach importance to the close relationship through relationship investment (Sung and Kim, 2014; Van Vaerenbergh and Orsingher, 2016; Yue et al., 2020). According to Harrison-Walker (2019), the more an organization invests in the customer service relationship means the more importance attached to the relationship. However, most of the previous studies on relationship investment focused on the connection between the organization and external customers, but rarely touched on the investment in the internal relationship between the organization and internal employees (Hewagama et al., 2019; Huang, 2020). Internal relationship investment (IRI) can be defined as the tangible resources provided to employees to assist in completing tasks and work by the company in the purpose of enhancing the exclusive relationship between the company and employees. When organizations gain the importance of employees, they invest in internal relationships (Onkelinx et al., 2016; Mihardjo et al., 2020), Then, whether it plays an impact on the relationship quality improved by internal service recovery of the organization should be a topic for discussion that needs to be deepen (Thelen and Yue, 2021).

Furthermore, the support provided by internal service recovery and internal relationship investment is deployed by the leaders from all departments in the organization. During support deployment, a series of past interactions between the leader and the employees will exert an influence, among which the interactive relationship between the leader and the employees can be discussed with Leader-Member Exchange (LMX) (Audenaert et al., 2016; Newman et al., 2017). Garg and Dhar (2017) argued that leaders usually develop different exchange relationships with employees, covering from low-quality exchange relationship which is unidirectional from top to bottom, to high-quality relationship that is reciprocal and bidirectional (Audenaert et al., 2019). In the case of unequal support derived from different exchange relationships, notwithstanding that the organization has turned to internal service recovery, will the effectiveness of remedial measures be impacted if employees perceive unequal distribution? Therefore, the study also intends to figure out whether the internal relationship quality produced by the internal service recovery of organizations will be different in the case of different exchange relationships.

In addition to the differences caused by the epidemic, the cross-cultural perspective can be seen as an important moderator that upholds individual feelings and independence (Rehg et al., 2012; Meyers et al., 2019; Lee et al., 2021; Zhao et al., 2021). There are differences in the development of employees’ internal relationship quality in the context of different culture, even in Asian regions (Shogren et al., 2018; Al-Jubari et al., 2019). Hence, employees from Mainland China and Taiwan were used as samples for a cross-cultural comparative analysis in this study to explore the regional differences in working activities (Meyers et al., 2019). Therefore, this study focused on determining employees’ perceptions of internal service recovery, internal relationship investment, LMX and internal relationship quality.

According to the above arguments, this study aims to provide the following contributions: (1) employees’ internal service recovery is explored from the perspective of internal marketing; (2) the effect that comparisons of cross-cultural effect bring to conceptual framework is discussed; (3) the verification of relationships among the internal service recovery, internal relationship investment, LMX and internal relationship quality is discussed, and managerial implications are provided for Asian companies. This study is organized as follows. In Section 2, we present a literature review and hypotheses development, while Section 3 presents the methodology and Section 4 explains the results of statistical analysis. Section 5 presents the conclusions.

## Literature Review and Hypotheses Development

### Internal Relationship Quality

Relationship quality means to establish a good relationship with customers by means of relationship marketing, so as to reduce customers’ uncertainty about the deal (Al-alak, 2014; Jelodar et al., 2016). Despite relevant studies indicate that the internal relationship is quite significant, there is a lack of discussion of organization-employee relationship in the literature on relationship marketing (Kim and Rhee, 2011; Lee and Kim, 2017, 2020), especially from the perspective of employees (Huang, 2020). As internal marketing develops, Huang (2020) believed that relationship marketing orientation not only means to establish a long-term relationship with customers, but also places emphasis on the importance of building a good relationship with employees to meet the needs of customers (Joung et al., 2015; Sarker and Ashrafi, 2018). Kim et al. (2016) and Rony and Suki (2017) indicated that a successful organization gain their competitive advantage through establishing relationships with their employees. Based on the spirit of internal marketing, the study regards employees as internal customers, transforms relevant theories of relationship quality into internal relationship quality, and defines it as the result of various positive relationships between the organization and the employees (Joung et al., 2015; Lee and Kim, 2020). In conformity with the measurement model proposed by scholars, organizational trust, organizational commitment and job satisfaction (Beloucif et al., 2004; Lee and Kim, 2020) are taken as the measurement variables of internal relationship quality.

Trust, which is generally viewed as an essential factor for successful relationships (Morgan and Hunt, 1994), is also an issue that is often discussed and valued. Trust is a psychological state, which means employees’ perception of the reliable overall assessment of the organization (Lee and Kim, 2020). In regard to employees’ trust in the organization, Wang and Hsieh (2013) considered that after conducting overall assessment on the organization, employees agree with the organization’s policies and principles, and are willing to expose themselves to vulnerable situations when they are unable to monitor the organization. Job satisfaction refers to employees’ intuitive feelings about their work and their physiological and psychological perceptions of satisfaction to environmental factors. Hewagama et al. (2019) considered that job satisfaction refers to the emotional and perceptual response to the gap between the actual and expected remuneration of the work itself and the work environment produced by workers (Lee and Kim, 2020; Qing et al., 2020), including internal satisfaction (such as senses of achievement, self-esteem, autonomy, feedback, control, etc.) and external satisfaction (affirmation and praise from supervisors, harmonious relationships among colleagues, good work environment, welfare, high salary, promotion, etc.) (Joung et al., 2015; Sarker and Ashrafi, 2018). Through job satisfaction, organizations can figure out as soon as possible whether employees suffer from inappropriate deployment at work and whether there is a lack of strategies or plans, so that proper remedial measures can be taken as an important reference for organizations to formulate strategies (Lambert et al., 2016; Rony and Suki, 2017). Mihardjo et al. (2020) indicated that organizational commitment refers to a kind of positive attitude toward the organization from employees. Employees identify with the organization’s goals, who are convinced to consider their work as part of their daily life and produce loyal and emotional belonging, thus they are willing to stay in the organization (Joung et al., 2015; Lee and Kim, 2020; Qing et al., 2020). Lee et al. (2006) deemed that organizational commitment serves as the main element of the fulfillment, innovation and stability of organizational goals, which can facilitate the relationship between managers and subordinates (Lambert et al., 2016), and improve organizational climate. As organizational commitment increases, the development, growth and survival of organizations can also be enhanced (Kim et al., 2016).

### Internal Service Recovery

Many scholars have illustrated that satisfaction obtained by customers with effective service recovery may be higher than satisfaction obtained during the first service (Van Vaerenbergh et al., 2019). Van Vaerenbergh and Orsingher (2016) argued that service failures are inevitable, but customer dissatisfaction can be avoided. Schlesinger and Heskett (1991) explained a series of reactions which occur when a service failure appears from the perspective of cycle, totally including two cycles: customer cycle and employee cycle (Park and Tran, 2018). First, customer cycle means that the customer dissatisfaction led by service failure will make the organization fail to establish a sustainable relationship with customers, thus resulting in a failure to cultivate customer loyalty and continuously producing dissatisfied customers (Harun et al., 2018; Harrison-Walker, 2019). Second, employee cycle means that dissatisfied employees (poor service attitude) will lead to high employee turnover (Van Vaerenbergh and Orsingher, 2016; Hewagama et al., 2019). Thus, narrow work design is made to put employees in a lack of competence of dealing with customer problems (Huang, 2020). However, the two cycles are not independent of each other, but have mutual effect (Mihardjo et al., 2020), and any link in any cycle can affect the process of the service. Thereby, during providing services, the organization must not only take into account the psychological feelings from customers, but also meet the job satisfaction of internal customers (employees) (Sarker and Ashrafi, 2018; Hewagama et al., 2019).

Most of the previous literature on service recovery focused on external service recovery (Van Vaerenbergh and Orsingher, 2016; Harun et al., 2018; Harrison-Walker, 2019; Van Vaerenbergh et al., 2019), but little touched on internal service recovery for internal customers. Nevertheless, Bowen and Johnston (1999) stated that remedial measures are necessary for both internal and external customers when a failure occurs, as the implementation of internal service recovery affects the implementation of external service recovery (Hewagama et al., 2019; Mihardjo et al., 2020). The construct of internal service recovery was proposed by Bowen and Johnston (1999), of which the main meaning is (1) reducing employees’ negative feelings when dealing with customer complaints, and (2) making employees more confident in providing services to satisfy customers in the future (Park and Tran, 2018; Sarker and Ashrafi, 2018). Acquiring the needs and expectations of employees is quite important for administrators, especially in the process of service delivery, where the performance of front-line employees determines the success of the service business (Yoo et al., 2006; Park and Tran, 2018; Mihardjo et al., 2020). Bowen and Johnston (1999) studied and believed that the way of external service recovery is also applicable to internal service recovery for front-line employees. Administrators can implement internal service recovery through effective internal marketing measures.

In this study, internal service recovery is defined as follows: the organization is aware that employees usually need to face the helplessness derived from service failures at work, thus a set of complete internal service recovery system is in need to keep down the sense of incapability possibly produced by employees, so as to support employees in dealing with external service recovery. Bowen and Johnston (1999) considered that four methods can be adopted for internal service recovery, namely empowerment, social support, employee involvement and reward (Sarker and Ashrafi, 2018). Thereby, the four dimensions are taken as strategies for internal service recovery in this study.

*Empowerment* endows employees with freedom to make decisions, providing situations in which employees make decisions in their daily work (Hewagama et al., 2019). Ergeneli et al. (2007) also indicated that empowering employees not only enables front-line employees to make direct response to customer needs during service delivery, but also to deal with dissatisfied customers immediately in the event of service failures. Moderate empowerment provided by the organization will exert a positive impact on the attitudes and behaviors from those who directly contact with customers. Service employees who are empowered will have more autonomy and flexibility, and in addition to being more satisfied with their work (Hewagama et al., 2019; Qing et al., 2020), their commitment to the organization can even be enhanced. Moreover, empowerment can also reduce the pressure while working, so as to increase the confidence of task implementation and enhance the capability to adapt to service changes (Chebat and Kollias, 2000; Liu et al., 2019). Qing et al. (2020) investigated the effect of ethical leadership on employee attitudes (affective commitment and job satisfaction), they found that psychological empowerment can effectively improve employees’ affective commitment and job satisfaction. Thereby, when employees feel that proper empowerment provided by the organization can reduce their sense of incapability, motivate them to increase work efficiency, facilitate service quality and improve customer service, then they will believe that the organization shows sincere care for them, thus increasing employees’ trust, organizational commitment and job satisfaction (Lee et al., 2006; Qing et al., 2020).

*Social Support* refers to the emotional support, information support and work support that the organization offers to employees to contribute to problem solving and enhancing the capability of resolving problems for employees (Kim et al., 2016; Lambert et al., 2016). Organizations agree that employees are entitled to making change, and they encourage employees to take responsibility for what they think they are competent to do (Sarker and Ashrafi, 2018), and make front-line employees perceive of respect and acceptance from organizations to gain satisfaction, thus producing trust, emotions and belonging to organizations. Chow et al. (2006) even argued that providing proper social support to employees can not only strengthen their customer-oriented behavior, but also increase their satisfaction with the organization. Lambert et al. (2016) indicated that employees feel social support from the organization, it contributes a great to relieving the pressure arising from the occurrence of service failures. Thus, organizations must provide front-line employees with love and care, as well as assistance and feedback of various emotions, materials, tools and information, to help employees solve problems they encounter and improve their capability of resolving problems (Lambert et al., 2016; Sarker and Ashrafi, 2018). Therefore, social support is conducive to improving employees’ confidence in completing work and perception of mutual care, and employees will produce feelings other than obligations, thus enhancing organizational commitment and trust.

*Employee Involvement* refers to the degree of participation and autonomy when employees engage in work (Raub, 2017), such as the degree of communication and information sharing, and the right to participate in decision-making (Diefendorff et al., 2002; Adham, 2014). From the perspective of employee perceived control, the greater the perceived control, the higher the employees’ job satisfaction, organizational commitment, level of involvement, performance and work motivation (Budd et al., 2010; Amah and Ahiauzu, 2013). Robbins et al. (2002) considered that psychological involvement will affect the continuous loss of employees in the work environment, positively enhance their perception and attitude, and improve their morale and organizational commitment (Bhatnagar, 2005; Amah and Ahiauzu, 2013). Besides, it is available for organizations to know the opinions and views raised by employees through employee involvement (Budd et al., 2010; Raub, 2017), satisfy employees’ expectation for decision-making control and endow employees with the right to participate in decision-making to improve their cognitive control (Diefendorff et al., 2002), thus improving employees’ organizational trust, organizational commitment and job satisfaction.

*Reward* means all formal management mechanisms and policies that an organization can provide and reward employees for their behavior and performance (Joung et al., 2015). The occurrence of service failures may produce “learned helplessness” for employees, thus organizations necessarily establish a set of perfect internal service recovery system under the concept of internal marketing to reduce the possibility that employees feel helplessness (Sarker and Ashrafi, 2018; Mihardjo et al., 2020), so that employees will show more confidence while providing services to external customers. Lee et al. (2006) deemed that employees’ behavior will be influenced by explicit and specific reward schemes. If employees can gain reward from their work performance, they will have a high level of job satisfaction and organizational commitment (Joung et al., 2015). Thereby, through a remedial regime, employees can feel that the organization is reliable, and their satisfaction with the work content provided by the organization will be improved (Joung et al., 2015; Mihardjo et al., 2020), and they are willing to strive for a long-term relationship. Therefore, the following hypothesis was proposed in this study:

H1: Internal service recovery has a positive and significant impact on internal relationship quality.

### Internal Relationship Investment

Scholars have indicated that relationship investment is the resources invested by firms for commitment to mutual relationships, among which there are extrinsic resources (expenditures of time, money and resources) and intrinsic resources (emotional investment, shared understanding and events accompanied by relationships) (Sung and Kim, 2014; Yue et al., 2020). From the perspective of resource-based theory, the relationship investment in internal customers (employees) within an organization can be regarded as a kind of investment in human capital (Onkelinx et al., 2016; Thelen and Yue, 2021). According to Galunic and Anderson (2000), human capital refers to the know-how, information, relationships and general abilities that employees will provide to the organization through their relationships with the organization, thus influencing the interests for the organization (Uppal and Mishra, 2014; Liguori et al., 2019). If an organization recognizes the importance of employees, it will engage in internal relationship investment, make designs for work design, organizational culture and organizational vision, market to employees and achieve customer satisfaction through employee satisfaction (Ahmed and Nawaz, 2015; Akgunduz et al., 2018). Therefore, in addition to investing resources to maintain the relationship with external customers, organizations must also invest resources into the organization to keep the specific relationship between the organization and internal customers (employees) (Thelen and Yue, 2021). In addition, the internal relationship investment input by the organization can also increase the conversion cost of employees, thus accelerating their dependence on the organization. If an organization increases investment in human capital, it will facilitate employees to invest their own abilities and knowledge, which will not only improve organizational capacity and performance, but also promote the relationship between employees and the organization.

Galunic and Anderson (2000) deemed that, despite internal relationship investment will reduce the elasticity of the organization, it can make employees feel the commitment to the relationship made by the organization, implying that the organization will make a guarantee to maintain and develop the relationship (Ahmed and Nawaz, 2015). Hitt et al. (2001) also found that investment in human resources can enhance the relationship between employees and the organization, as well as promoting employee involvement (Uppal and Mishra, 2014; Onkelinx et al., 2016; Liguori et al., 2019). It is also found that when employees perceive the willingness of the organization to strive for the relationship between them, employees are able to get involved in the remedial measures implemented by the organization and believe that the policies from the organization are good for them. Thus, the positive impact of internal service recovery measures on internal relationship quality can be enhanced (Thelen and Yue, 2021). Therefore, the following hypothesis was proposed in this study:

H2: Internal relationship investment positively moderates the relationship between internal service recovery and internal relationship quality.

### Leader-Member Exchange

Leader-member exchange represents the quality of social exchange relationship between supervisors and subordinates (Graen, 1976; Graen and Scandura, 1987), of which one of the elements is interpersonal trust between the two interactive sides (Scandura and Pellegrini, 2008). Based on the principle of social exchange, no matter materially or spiritually, leaders will treat the members within the in-group in a special way (Xu et al., 2012; Newman et al., 2017), such as assigning challenging tasks and providing greater power to make decisions, as well as expecting them to complete tough tasks for themselves, or taking on more responsibilities or requiring to undertake informal roles (Audenaert et al., 2016). In-group members are not only regarded as one of their own, but also receive more attention and support, and are allocated more resources and benefits, thus they have higher job satisfaction (Peng et al., 2014; Garg and Dhar, 2017; Audenaert et al., 2019). For out-group members with low exchange quality, the essence of interaction with leaders tends to be formal work contract (Xu et al., 2012; Peng and Lin, 2016), and subordinates perform only the tasks specified in the job description in line with the role specified in the work contract and maintain the principle of economic exchange with leaders (economic exchange) (Landry and Vandenberghe, 2009). Thus, the exchange quality between in-group members and leaders is quite good, and there exists a high level of relationship in mutual trust, mutual loyalty, mutual respect and mutual obligation (Sparrowe et al., 2006; Garg and Dhar, 2017; Audenaert et al., 2019). Comparatively, out-groups with lower LMX relationship have less negotiating latitude, mutual respect and mutual loyalty. Dominance-like communication and autocratic decision making are preferred between employees and leaders (Sparrowe et al., 2006; Garg and Dhar, 2017).

When there is a high-quality LMX between the leader and the employees, the employees can be more accessible to the resources entrusted by the leader. When the organization implements remedial strategies and measures, the employees are more willing to cooperate with the organization, cultivate trust and organizational commitment, thus improving job satisfaction (Audenaert et al., 2016; Newman et al., 2017). However, for the leader, employees with low-quality LMX belong to an out-group, and there exists only a formal and contractual relationship between them (Yu and Liang, 2004; Xu et al., 2012; Peng and Lin, 2016), making the employees produce a dubious attitude toward the organization. Hereon, the perceived fairness of the resources allocated to employees or during the implementation of decisions will exert an impact on job satisfaction, and the higher the awareness of inequity is, the lower the trust in the leader will be (Garg and Dhar, 2017; Audenaert et al., 2019). At this point, the trust between the leader and the employees will weaken, and employees will certainly have low commitment behavior to the organization (Nyhan, 1999; Audenaert et al., 2019). When the organization proposes remedial strategies or measures, employees with low-quality LMX are less involved in the remedial measures than employees with high-quality LMX, thus resulting in a failure to gain the effectiveness of remedial measures, and relatively weakening the internal relationship quality between employees and the organization. Therefore, the following hypothesis was proposed in this study:

H3: The degree of LMX quality moderates the relationship between internal service recovery and internal relationship quality, and employees with high-quality LMX can enhance the relationship between internal service recovery and internal relationship quality, but employees with low-quality LMX show a poorer effect.

**FIGURE 1:**
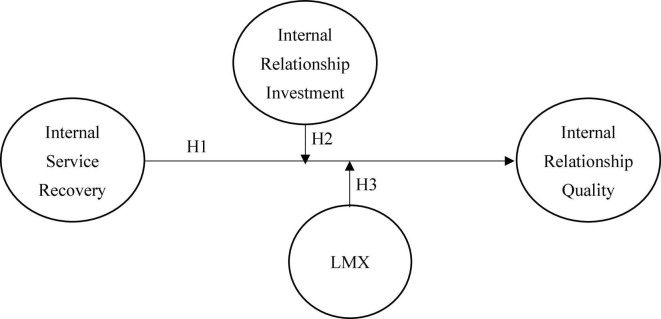
Conceptual framework.

Based on the above research motivation and the research significance, as outlined in the plan, we aimed to verify the following research framework, shown in Figure 1.

## Methodology

### Sampling

This study aims to explore the relationship between internal service recovery and internal relationship quality, and takes Leader-Member Exchange and internal relationship investment as moderators. In the study, it is believed that in addition to the service industry, the manufacturing industry also owns a large scale and a mature management and administration mode, which is equipped with a concept of certain internal marketing culture and perfect internal service recovery. Thus, the research object of this study is mainly the front-line employees in service and manufacturing enterprises who have a direct face-to-face opportunity with consumers or customers, and it conducts a discussion on the internal service recovery. In addition, the study also explores the differences when organizational administrators treat their employees in the case of different management cultures in Taiwan and mainland China. Before handing out the questionnaire, 40 copies of questionnaire were first handed out for the pretest in this study. After confirming the reliability of the items in the scale, a formal follow-up test was carried out. A total of 800 copies of questionnaire were distributed in this study, of which 400 copies were sent to Taiwan and 400 copies were sent to local enterprises of Hangzhou and Shanghai in mainland China. As for the samples of Taiwan, a total of 260 copies of questionnaire were collected, of which 10 copies were invalid and 250 copies were valid, with a recovery rate of 62.5%. In regard to the samples of mainland China, a total of 206 copies of questionnaire were collected, of which five copies were invalid and 201 copies were valid, with a recovery rate of 50.2%. In the Taiwanese sample, most are male (65.3%), whose level of education is mostly undergraduate or above (80.4%), and most of them are between 30 and 40 years old (64.5%) with average working year of 3.5. In the sample of mainland China, most are male (58.4%), whose level of education is mostly undergraduate or above (63.3%), and most of them are between 30 and 35 years old (44.8%) with average working year of 4.8.

As there may be a reaction bias between the early and later samples, the questionnaires were divided into two groups, early and later, according to the suggestions from Armstrong and Overton (1977), and they were tested whether there were significant differences between the two groups in the sample data and the dimension. Verification results show that there is no significant difference between the main dimensions and the basic information, which indicates that the problem of possible bias from non-response bias is not serious.

This study hid the names of constructs, and assigned the question items randomly to prevent common method variance (CMV). The Harman one-factor analysis method as used to test for CMV. The explained variance in one factor was 41.39%, which is smaller than the recommended threshold of 50%. Therefore, CMV was not problematic in this study (Podsakoff and Organ, 1986).

### Measures

With the concept of internal marketing, taking the views from Smith and Bolton (1998), Wulf et al. (2001), Roberts et al. (2003), and Beloucif et al. (2004), on the formation of the constructs for relationship quality, it is considered that relationship quality reflects the appropriateness of meeting employees’ needs and expectations through the three constructs of organizational trust, job satisfaction and organizational commitment.

Based on the study by Bowen and Johnston (1999), internal service recovery believes that external service recovery is also applicable for internal recovery for front-line employees. Thereby, administrators can facilitate internal service recovery through effective steps of external service recovery, which mainly includes four key steps: response, information, activities and compensation, and it explains that internal service recovery can be achieved mainly through four methods: empowerment, social support, employee involvement and reward.

Internal relationship investment refers to the tangible and intangible resources that employees feel the organization pays to maintain the relationship. In terms of variable manipulation, this study refers to the study made by Galunic and Anderson (2000), including seven items. Leader-Member Exchange theory is defined as the inner cognition degree of the relationship between employees and supervisors, and refers to the scale developed by Graen and Uhl-Bien (1995), which includes eight items.

## Results

### Measurement

All latent variables evaluated were found to be reliable in this study, with Cronbach’s α ranging from 0.837 to 0.946. Table 1 shows the reliability of each scale, the reliabilities in each latent variables have been good, with a Cronbach’s α over 0.70. In order to verify validity of measurement model, this study conducted confirmatory factor analysis (CFA) *via* PLS-SEM to examine the construct validity, including convergent and discriminant validity. Based on validity criteria recommended from Hair et al. (2010), CFA results show that standardized factor loadings were higher than 0.5; average variance extracted (AVE) ranges between 0.792 ∼ 0.920; and composite reliability (CR) ranges between 0.921 ∼ 0.983. All three criteria for convergent validity were met, and correlation coefficients were all less than the square root of the AVE within one dimension, suggesting that each dimension in this study had good discriminant validity.

**TABLE 1:** Measurement.

	Empowerment	SS	EI	Reward	OT	OC	JS	LMX	IRI
Empowerment	*0.890*								
SS	0.489	*0.940*							
EI	0.519	0.689	*0.944*						
Reward	0.346	0.307	0.397	*0.956*					
OT	0.429	0.775	0.722	0.437	*0.959*				
OC	0.414	0.659	0.666	0.384	0.804	*0.918*			
JS	0.486	0.633	0.675	0.532	0.730	0.770	*0.946*		
LMX	0.436	0.742	0.783	0.392	0.795	0.742	0.702	*0.920*	
IRI	0.435	0.662	0.682	0.505	0.750	0.693	0.732	0.807	*0.901*
α	0.863	0.946	0.915	0.837	0.899	0.837	0.883	0.931	0.885
AVE	0.792	0.884	0.891	0.914	0.920	0.843	0.894	0.846	0.812
CR	0.921	0.942	0.945	0.955	0.983	0.947	0.962	0.959	0.922

*Italic values are square root of AVE for each latent construct in diagonals.*

### Inner Model Analysis

**FIGURE 2:**
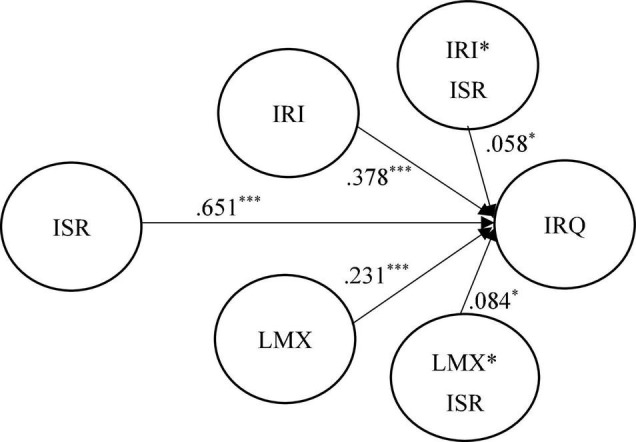
Structural model on Taiwanese employees. *p < 0.05 and ^***^p < 0.001.

**FIGURE 3:**
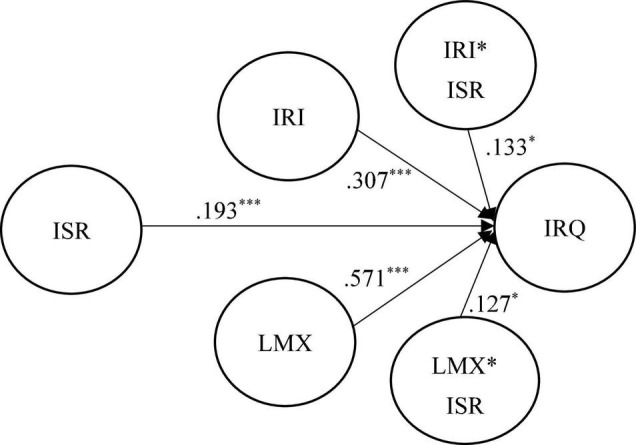
Structural model on mainland China employees. *p < 0.05 and ^***^p < 0.001.

To assess the structural model, Figures 2, 3 and Tables 2, 3 show the results of the hypothesized relationships and standardized coefficients in Mainland China and Taiwanese samples. The results showed that internal service recovery was positively and significantly related to internal relationship quality (β_Mainland China_ = 0.193, *f*^2^ = 0.331, *p* < 0.001; β_Taiwan_ = 0.651, *f*^2^ = 0.243, *p* < 0.001), supporting H1. The greater the high degree of internal service recovery, the employees are willing to maintain the closer relationship with organizations. Research results showed that internal relationship investment was positively and significantly moderating relationship between internal service recovery and internal relationship quality in both regions (β_Mainland China_ = 0.133, *f*^2^ = 0.016, *p* < 0.05; β_Taiwan_ = 0.058, *f*^2^ = 0.012, *p* < 0.001), supporting H2. The results indicate that the higher degree of internal relationship investment makes employees feel more about internal service recovery provided by the company, and establish a strong internal relationship quality.

**TABLE 2:** Measurement of scale.

Construct	Variables	Items
Internal Service Recovery	Empowerment	The supervisor supports and agrees with the way I conduct service recovery.
		I have the opportunity to be fully independent and autonomous in work execution.
		When customers are dissatisfactory with my service, the organization allows me to make an independent judgment and offer the best service recovery.
	Social support	The organization is willing to give ear to my troubles at work.
		When I am down in spirits due to work, the organization will provide spiritual support.
		The organization shows concern about the work troubles I encounter.
		When troubles occur in my work, the organization will provide me with proper advices and suggestions.
		The organization provide me with some substantial help and support to solve my work stress.
	Employee involvement	When it comes to relevant decisions, I can participate and express my opinions and views.
		The organization will consider or adopt my advice to prevent internal service failures.
		The organization will ask for my advices and opinions on improving service failures.
		The organization will ask for my advices before implementing new policies or measures.
	Reward	The organization will duly offer me a financial reward (a small gift, monetary reward or job bonus).
		The organization will duly offer me non-financial rewards (job promotions, important task assignments and public praise).
Internal Relationship Quality	Organizational trust	I believe the organization shows genuine care for me.
		I believe the organization would not withhold information I need to know.
		I believe the organization would give priority to my interests.
	Job satisfaction	I feel satisfactory with the work contents provided by the organization.
		I feel satisfactory with the work environment provided by the organization.
		I feel satisfactory with the promotion system, welfare system and salary system provided by the organization.
		On the whole, I am satisfactory with the organization.
	Organizational commitment	I take the problems that the organization encounter as my own.
		I feel a strong sense of belonging to the organization.
		I will stay with my current organization as other organizations cannot offer me a better salary.
		Staying on with the organization is a necessity for me.
LMX	LMX	I know how satisfied my immediate supervisor is with my work.
		I know my immediate supervisor can know my work problems and needs.
		I consider that my immediate supervisor treats me as “a potential employee.”
		When I encounter problems at work, my immediate supervisor wields his authority to help me solve it.
		When I need it, my immediate supervisor will assist me at their own expense.
		I have full confidence in my supervisor and I will defend his decisions.
		I think I have good friendly sentiments with my immediate supervisor.
Internal Relationship Investment	Internal Relationship Investment	While I am dealing with work, the organization can provide available help.
		I often apply the organization’s consulting services to getting through my work.
		The organization gives me advice on how to get through the work.
		The organization will subsidize my office expenses.
		The organization will provide a grand so that I can accomplish my work smoothly
		If I ask for a grant, the organization will approve my application.

**TABLE 3:** Results of the hypotheses testing.

Paths	Mainland China		Taiwan		Decision
	β	*p*-value	β	*p*-value	
H1: ISR → IRQ	0.193	0.000	0.651	0.000	Support
H2: ISR × IRI → IRQ	0.133	0.022	0.058	0.048	Support
H3: ISR × LMX → IRQ	0.127	0.031	0.084	0.027	Support

**FIGURE 4:**
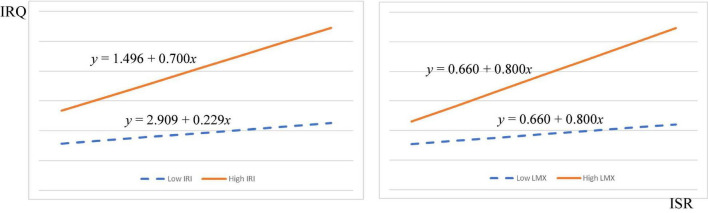
Interaction diagram of IRI and LMX between ISR and IRQ in mainland China.

**FIGURE 5:**
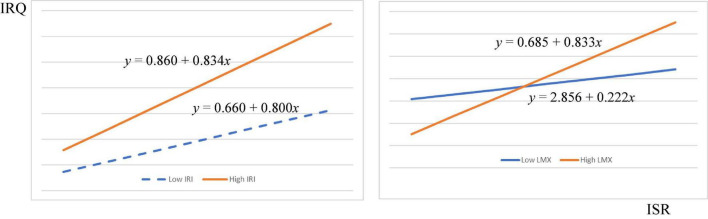
Interaction diagram of IRI and LMX between ISR and IRQ in mainland China.

Moreover, LMX (β_Mainland China_ = 0.127, *f*^2^ = 0.112, *p* < 0.001; β_Taiwan_ = 0.084, *f*^2^ = 0.032, *p* < 0.05) was also moderating relationship between internal service recovery and internal relationship quality in both regions, supporting H3. Figures 4, 5 exhibit the interaction of IRI and LMX on the relationship between ISR and IRQ. The graph shows that if ISR practice more IRI and LMX with concerned parties, the moderation effects will be higher, and IRQ will be enhanced.

## Conclusion

### Discussion

Service failures are inevitable, and there has been considerable studies on the concept of service recovery literature (e.g., Van Vaerenbergh and Orsingher, 2016; Harrison-Walker, 2019; Hewagama et al., 2019; Mihardjo et al., 2020). However, most of them are conducted from the perspective of customers, and rarely discusses service failures and the remedial effectiveness made by the organization from the perspective of employees (Hewagama et al., 2019). Through the application of internal marketing concept, this study intends to discuss the relationship between internal service recovery and internal relationship quality from the perspective of internal customers, and takes internal relationship investment and LMX as moderators, so as to know the resources and leadership factors that enhance internal service recovery.

It is concluded by the study that internal service recovery exerts a positive impact on internal relationship quality. The results show that internal service recovery has a positive and significant impact in both mainland China and Taiwan. The result is similar to that from previous studies that if an organization has adequate resources to implement psychological compensation or enhancement schemes for employees, it will contribute to cultivating employees’ cohesion to the organization and internal relationship (Yoo et al., 2006; Park and Tran, 2018; Sarker and Ashrafi, 2018; Hewagama et al., 2019; Mihardjo et al., 2020). In the literature, few studies have examined internal service recovery as an important antecedent in internal marketing, linking strategic human resource management to employees’ positive psychological status and attitudes to organization (Sung and Kim, 2014; Van Vaerenbergh and Orsingher, 2016; Yue et al., 2020). After Bowen and Johnston (1999) proposed the concept of internal service recovery, until 2006, Yoo et al. adopted an exploratory method to demonstrate the key elements of the internal service recovery strategy by taking the front-line employees in the catering industry as the research objects. As the research on internal service recovery is still in the stage of theoretical development, the studies on internal service recovery are relatively few and mostly exploratory. Hence, this study explores the important role that internal service recovery plays in organizations by means of empirical research, and further expands relevant research on internal service recovery.

Furthermore, this study also illustrates the importance of internal relationship investment with the concept of internal marketing. Most previous studies focused on the relationship investment of external customers (Sung and Kim, 2014; Van Vaerenbergh and Orsingher, 2016; Yue et al., 2020), rarely applied relationship investment to internal employees within the organization (Galunic and Anderson, 2000; Thelen and Yue, 2021). In addition, this study also has confirmed that internal relationship investment plays an invigorating effect on internal service recovery and improve the internal relationship quality between the organization and employees. Harrell-Cook and Ferris (1997) also believed that a lack of investment in human resources will result in reduced social legitimacy of organizations, so that the ability for organizations to effectively recruit and retain employees can be influenced. Thus, attracting and retaining highly skilled and knowledgeable employees is essential to an organization’s long-term competitive advantage (Onkelinx et al., 2016). Therefore, the results provide strong importance for internal relationship investment.

Besides, the study believes that the relationship between leaders and employees will affect the effectiveness while organizations are implementing internal service recovery. The results also have confirmed that employees who have a good relationship with the leader have a higher acceptance of organizational policies than those who have a bad relationship, which also meets the goal the organization intends to achieve (improving internal relationship quality). The findings are consistent with the claims from Sparrowe et al. (2006), Garg and Dhar (2017), and Audenaert et al. (2019) that employees with high-quality LMX will enter into high-level relationship interaction from social exchange and have positive views on the leadership style and the human resource practice of organizations. Leader-Member Exchange (LMX) was regarded as a positive antecedent variable or a positive result in most previous studies (Audenaert et al., 2016; Garg and Dhar, 2017), but few studies have examined the negative effects of Leader-Member Exchange as a variable in an intervening model (Audenaert et al., 2019). Thus, the significant findings of this study contribute to the leadership theory development of LMX, and the theoretical model in this study can provide a reference for relevant research in the future.

### Practical Implications

Based on the results from this study, several pieces of suggestions are provided for practical reference. Firstly, the results show that internal service recovery exerts a significantly positive impact on internal relationship quality, indicating the importance of internal service recovery strategy for the organization. The study suggests that administrators need to provide a perfect internal service recovery mechanism to support employees’ efforts in external service recovery, so as to improve their organizational commitment. Thereby, enterprises should attach importance to the issue of internal service recovery. Secondly, the jamming effect of internal relationship investment has a significant effect, indicating that organizations should make efforts to maintain a relationship with employees in usual, instead of taking remedial measures when the employees themselves are warned of negative work quality. In this study, it is considered that human capital is what any organization needs to establish and maintain. To make employees willing to pay and get involved in the organization, part of tangible or intangible resources ought to be gradually invested to guide employees’ centripetal force to the organization. Through the implementation of internal service recovery mechanism, it will be more effective and complete than establishing a good internal relationship with employees only by means of internal service recovery mechanism.

Finally, the jamming effect of LMX exerts a significant impact, showing that in addition to establishing strategies to maintain the relationship with employees, organizations also need to take the relationship between administrators and employees into consideration. This study suggests that administrators should deeply understand whether the internal bidirectional communication channels, performance and compensation system are sound, and strengthen the function of eliminating relationships and privileges in organizational justice. If these employees fail to acquire more resources due to their good relationships with administrators or special background, it may increase the fatigue of other employees, produce negative work quality, make employees less willing to actively accept the policies promoted by organizations, and result in effect reduction.

### Research Limitations

The relevant research on internal service recovery discussed in this study is a developing management issue, thus it still lacks mature reference dimensions to comply. Thus, the completeness and representativeness of relevant literature data collected in this study are relatively limited, and for research issues which are developing, its variability research is relatively improved. In terms of subsequent related research, different and multi-faceted research dimensions for discussion are suggested to make the inferential basis of the research more rigorous and representative. In addition, as for the literature on service recovery, equity theory was applied to explore customer satisfaction after customers evaluated service recovery in the past. Thus, it is suggested that follow-up research can be conducted to further explore employees’ satisfaction with internal service recovery. Furthermore, previous studies have found that the relationship quality between organizations and employees and the relationship between organizations and customers have a positive correlation (Gummesson, 1999). Hence, the follow-up research can explore how effective internal service recovery affects external service recovery and then how to affect the relationship quality between employees and organizations, as well as the relationship quality between customers and organizations.

Besides, internal service recovery can be regarded as an important issue in internal marketing literature, but few studies have given prominence to the importance in the past. Notwithstanding that the study has planned a research model to compare the cognition and attitude of employees from Taiwan and Mainland China in the service industry, more sample sizes and different regions and countries are still needed to enrich the theoretical connotation of the concept. It is suggested in this study that subsequent researchers can add different industries or regions to enhance the generalization of the concept.

## Data Availability Statement

The raw data supporting the conclusions of this article will be made available by the authors, without undue reservation.

## Ethics Statement

The studies involving human participants were reviewed and approved by Ethics Committee of Foshan University. The patients/participants provided their written informed consent to participate in this study.

## Author Contributions

This study is a joint work of all authors. YL, MX, W-HC, and LL contributed to the ideas of educational research, collection of data, and empirical analysis. MX and W-HC contributed to the data analysis, design of research methods, and tables. LL and YL participated in developing a research design, writing, and interpreting the analysis. All authors contributed to the literature review and conclusion.

## Conflict of Interest

The authors declare that the research was conducted in the absence of any commercial or financial relationships that could be construed as a potential conflict of interest.

## Publisher’s Note

All claims expressed in this article are solely those of the authors and do not necessarily represent those of their affiliated organizations, or those of the publisher, the editors and the reviewers. Any product that may be evaluated in this article, or claim that may be made by its manufacturer, is not guaranteed or endorsed by the publisher.
